# Detection of *FMR1* CGG Repeat Expansions Using Buccal Swab and Blood Samples of Children With Intelectual Disability in A Resource-Limited Country^☆^

**DOI:** 10.1016/j.jgeb.2025.100582

**Published:** 2025-10-02

**Authors:** Siti F. Aulia, Mentari Amir, Intan Razari, Kinasih Prayuni, Wan Nedra, Ndaru A. Damayanti, Nurmayani Irwandi, Ahmad Utomo, Vivienne J. Tan, Samuel S. Chong, Sultana M.H. Faradz

**Affiliations:** a Graduate Student on Genetic Counseling, Biomedical Sciences, YARSI University, Jakarta, Indonesia; b Genomic Research Center, YARSI Research Institute, YARSI University, Jakarta, Indonesia; c Department of Paediatrics, Faculty of Medicine, YARSI University, Jakarta, Indonesia; d Department of Biomedical Science, Post Graduate School, YARSI University, Jakarta, Indonesia; e Department of Paediatrics, Yong Loo Lin School of Medicine, National University of Singapore, Singapore; f Department of Obstetrics and Gynaecology, Yong Loo Lin School of Medicine, National University of Singapore, Singapore

**Keywords:** Fragile X syndrome, Intellectual disability, *FMR1* gene analysis, Blood samples, Buccal swab samples

## Abstract

Fragile X Syndrome (FXS), the most common inherited intellectual disability, is caused by CGG-repeat expansions in the *FMR1* gene. In resource-limited settings such as Indonesia, the absence of systematic screening programs complicates early detection. This study compared the performance of buccal swab and blood samples in detecting *FMR1* repeat expansions to facilitate non-invasive screening. A total of 164 male students with intellectual disabilities in Jakarta provided paired buccal swab and blood samples. Conventional PCR was used for initial screening, followed by Triplet-Primed PCR (TP-PCR) with Melt Curve Analysis (MCA) and sizing confirmation by fluorescent TP-PCR and capillary electrophoresis. Conventional PCR identified 159 normal alleles, three grey zones, and two full mutations, resulting in an FXS prevalence of 1.22 %. A perfect concordance was observed between buccal swab and blood samples (Cohen’s Kappa = 1.000). Additional TP-PCR MCA analysis on 80 selected samples revealed inconsistencies in buccal swab results, with 12 cases classified as indeterminate, suggesting potential DNA quantity and/or quality issues. These findings indicated that buccal swabs are a feasible, non-invasive sampling method to screen FXS via conventional PCR, though further optimization is required for TP-PCR MCA. This study represents the first FXS screening in Jakarta, emphasizing the importance of early detection and scalable genetic testing strategies, particularly in resource-limited settings.

## Highlights

• Comparison of FXS testing using blood and buccal swab in children with ID.
• Conventional PCR and TP-PCR MCA methods applied to analyze CGG repeat expansion.
• Buccal swab shows promising concordance with blood in conventional PCR.
• Non-invasive sampling method offers feasible alternative for genetic screening.
• Study supports wider use of buccal swabs in FXS diagnosis among children with special needs.

## 1 Introduction

Fragile X syndrome (FXS) is one of the rare diseases, a condition caused by pathogenic variation in the *FMR1* gene (Fragile X Messenger Ribonucleoprotein 1) on the X chromosome due to expansions of the CGG trinucleotide repeat, affecting the function of the gene.^1^, ^2^ The prevalence of FXS is estimated to be approximately 1 in 7000 men, although the exact numbers are unclear.^3^

The number of CGG repeats within the *FMR1* gene determines the risk of fragile X-related conditions. Based on the repeat count, there are four primary categories: normal (up to 44 repeats), intermediate or grey zone (45–54 repeats), premutation (55–200 repeats) and full mutation (>200 repeats). Full mutations are strongly related to FXS, and clinical severity is influenced by factors such as methylation levels, sex, and mosaicism. Premutation alleles, while not typically associated with intellectual disabilities (ID), may lead to conditions such as Fragile X-associated tremor/ataxia syndrome (FXTAS) and Fragile X-associated primary ovarian insufficiency (FXPOI), particularly through maternal transmission.^1^, ^4^

Since the *FMR1* gene is located on the X chromosome at locus Xq27.3, males typically exhibit more pronounced symptoms of FXS compared to females.^5^, ^6^ Most males with FXS exhibit moderate to severe ID caused by the absence of Fragile X Messenger Ribonucleoprotein (FMRP).

Comprehensive and reliable data on the prevalence of FXS in Indonesia remain scarce. A study of high-risk populations in Central Java for two decades reported a prevalence of FXS of 0.9–1.9 % among children with identification in special schools in Central Java province, compared to 6.15 % among children with autism spectrum disorder (ASD).^7^ Despite this, there is no evidence of widespread FXS screening programs in Indonesia, including its capital Jakarta. In contrast, developed countries such as the United States and European countries have implemented systematic screening and diagnostic programmed for FXS, allowing earlier detection and intervention.^3^ This lack of screening and diagnostic efforts hampers early recognition and timely interventions, which are critical to improving learning outcomes and quality of life for affected individuals. The lack of comprehensive screening highlights a significant gap in public health initiatives, especially in a developing country with a large population like Indonesia. Polymerase chain reaction (PCR) and Southern blotting are the primary molecular techniques used to detect *FMR1* gene mutations in suspected FXS cases. Southern blotting remains the gold standard for confirming full mutations. However, conventional PCR alone is not sufficient for detecting full mutations, as conventional PCR struggles to amplify large CGG repeat expansions. With the advancement of newer PCR techniques, it is now possible to identify individuals with both premutations and full mutations.^8^ In addition, recent molecular advances have introduced long-read sequencing technologies such as PacBio and Oxford Nanopore (ONT), which allow for comprehensive characterization of CGG repeat expansions, methylation status, and mosaicism in a single assay.^9^ However, such technologies remain expensive and are not yet widely accessible in resource-limited settings.

As FXS is a leading genetic cause of ID, population screening of FXS would require obtaining samples from children with special needs. Invasive sample collection procedures such as drawing blood from peripheral veins of children with ID could be challenging to implement. Buccal swab sampling offers a non-invasive, cost-effective, and easily implementable method for genetic testing, making it highly suitable for use in children with special needs.

This study follows a stepwise approach, beginning with DNA extraction from buccal swab and peripheral blood samples, followed by conventional PCR for initial screening, which is then followed by triplet-primed PCR (TP-PCR) for more precise CGG repeat expansion detection. Conventional PCR can detect normal alleles and may indicate the presence of grey zone alleles, but it lacks the sensitivity to accurately distinguish premutations and full mutations.^10^ Therefore, TP-PCR combined with melting curve analysis (MCA) using high-resolution melting (HRM) technology is performed to classify CGG repeat expansions. This method categorizes individuals into normal allele (NL), intermediate (IM), and grey zone (GZ) groups, while also detecting premutations and full mutations when present. To precisely determine the number of CGG repeats, capillary electrophoresis is required, particularly for alleles in the premutation (55–199 repeats) and full mutation range (≥200 repeats).^11^

By integrating these innovative methods, this research aims to address the significant gap in FXS detection in Indonesia, a Resource-Limited Country. It not only strengthens early diagnostic precision, but also lays the groundwork for incorporating non-invasive genetic screening into public health infrastructure. These findings could serve as a model for other nations with limited resources, supporting early detection and improving outcomes for individuals with FXS and related conditions. Earlier screening efforts in Central Java, carried out by Faradz et al. (1995) and Sihombing et al. (2021), may not accurately reflect the nationwide prevalence of FXS in Indonesia. However, they remain valuable as supplementary data for understanding the presence of FXS within the country.

## 2 Materials and methods

The subjects of this study were male students with intellectual disabilities enrolled in 4 special schools in Jakarta. Inclusion criteria included male students with intellectual disabilities registered at Special Schools in Jakarta, whose parents or guardians provided informed consent. Exclusion criteria were female students with ID, students with Down syndrome, students with multiple congenital anomalies, or parents who declined to participate. A total of 164 subjects were initially recruited and 12 were excluded.

Blood and buccal swab samples were collected by trained medical personnel following standard medical procedures. Blood samples were collected using a sterile syringe and transferred to EDTA tubes. Participants were instructed not to eat or drink before collecting the buccal swab to minimize variability. Buccal swabs were collected using disposable sterile oral swabs (OneMed, Sidoarjo, Indonesia) which were gently rubbed on the inner cheek for 30 s per side, and stored in labelled tubes. To enhance documentation and reduce the possibility of sample mix-up, each subject was photographed along with their labelled tube after sample collection.

DNA extraction from whole blood collected with EDTA anticoagulant (5 ml) was carried out using the salting out method as describe in previous study (Faradz et al 1995). DNA extraction from buccal swab samples was performed using the Quick-DNA™ Miniprep Plus (ZymoResearch, California, USA) according to manufacturer’s biological fluid protocol. This kit was chosen for its high efficiency and consistent yield, which are crucial for obtaining sufficient DNA for subsequent analyses. The extracted DNA was stored at −20 °C until subsequent analysis.

Conventional PCR was performed using *FMR1* primers first described by Fu et al. (1991),^12^ with the following forward and reverse sequences, respectively: GCTCAGCTCCGTTTCGGTTTCACTTCCGGT and AGCCCCGCACTTCCACCACCA GCTCCTCCA. The PCR protocol was adapted with slight modification from Chen et al. (2017).^13^ The PCR mix contained 2.5 µL of 10 × PCR buffer, 2 µL of 2.5 mM dNTPs, 5 µL of Q solution, 0.75 pmol of each primer, and 1 unit of HotStar Taq DNA Polymerase (QIAGEN, Hilden, Germany).^12^ The cycling condition included an initial denaturation at 98 °C for 10 min, followed by 10 cycles of denaturation at 97 °C for 35 s, annealing at 64 °C for 2 min, and extension at 68 °C for 8 min. The subsequent 25 cycles were performed under similar conditions, and the extension step was prolonged to 8 min and 20 s per cycle. A final hold at 4 °C was applied at the end of the reaction. Post-PCR analysis was performed using 2 % agarose gel electrophoresis and DNA bands were visualized using a UV gel documentation system. The positive control was included in this study to validate the amplification result to detect CGG repeat alleles accurately. The positive control used in this study was the same as the sample studied by Faradz et al (1995).^12^ The inclusion of a positive control allowed verification of the assay's reliability in detecting known CGG repeat expansions, supporting the robustness of the PCR method used in this study.

Triplet-Primed PCR (TP-PCR) and Melt Curve Analysis (MCA) were performed to detect CGG repeat expansions in the *FMR1* gene according to Rajan-Babu et al., 2022.^14^ The MCA was performed using the Rotor-Gene Q 5plex HRM System (Qiagen, Hilden, Germany) to ensure consistent and accurate performance. The TP-PCR master mix contained genomic DNA, HotStar Taq DNA polymerase, dNTPs, *FMR1*-specific reverse primer f (5′-AGCCCCGCACTTCCACCACCAGCTCCTCCA-3′), Tail primer (5′-TGCTCTGGACCCTGAAGTGTGCCGTTGATA-3′), and TP primer (5′-TGCTCTGGACCCTGAAGTGTGCCGTTGATA[CGG]_5_-3′). The PCR cycling conditions involved an initial denaturation at 95 °C for 15 min, followed by 40 cycles of denaturation at 99 °C for 45 s, annealing at 55 °C for 45 s, and extension at 70 °C for 8 min. A final hold at 72 °C for 10 min was applied at the end of the reaction. MCA was carried out using Rotor-Gene Q with a slow temperature ramp from 65° C to 95° C to differentiate normal from expanded alleles based on melting peak shifts. The controls used were genomic DNA samples NA20232 (containing 46 CGG repeats) and NA20230 (containing 54 CGG repeats) purchased from Coriell Cell Repository (Camden, New Jersey).

Fluorescent TP-PCR and capillary electrophoresis (CE) were used to confirm expansion-positive samples and were conducted according to Rajan-Babu et al., 2022,^15^ except that capillary electrophoresis was conducted on the SeqStudio^TM^ Genetic Analyser (Applied Biosystems^TM^, Waltham, Massachusetts). For each sample, 1 µl of amplification product was mixed with 9 ul of Hi-Di^TM^ formamide (Applied Biosystems^TM^) and 0.2 µl GeneScan^TM^ 500 ROX^TM^ dye size standard (Applied Biosystems^TM^), denatured at 95 °C for 5 min before cooling to 4 °C. Samples were electrokinetically injected at 1.2 kv for 18 s and ran at 13 kV. GeneScan^TM^ analysis was performed using the Microsatellite Analysis application on Thermo Fisher^TM^ Cloud (Applied Biosystems^TM^). The presence of sharp high-quality fluorescent CE peaks was used to determine the exact repeat count. The CE process involved the analysis of PCR products to determine the exact size of CGG repeats, allowing differentiation between premutation and full mutation alleles. Samples were classified as expansion-positive based on specific threshold values for the size of the CGG repeat: premutation alleles (55–199 repeats) and full mutation alleles (≥200 repeats).

## 3 Results

The concentration of DNA varied between whole blood and buccal swab samples. In whole blood samples, DNA concentrations ranged from 20 to 600 ng/µl, while in the buccal swab samples, it ranged from 2 to 50 ng/µl. Despite the lower DNA concentration in buccal swabs, amplification was still detectable using conventional PCR, even at 2 ng/µl.

**Fig. 1:**
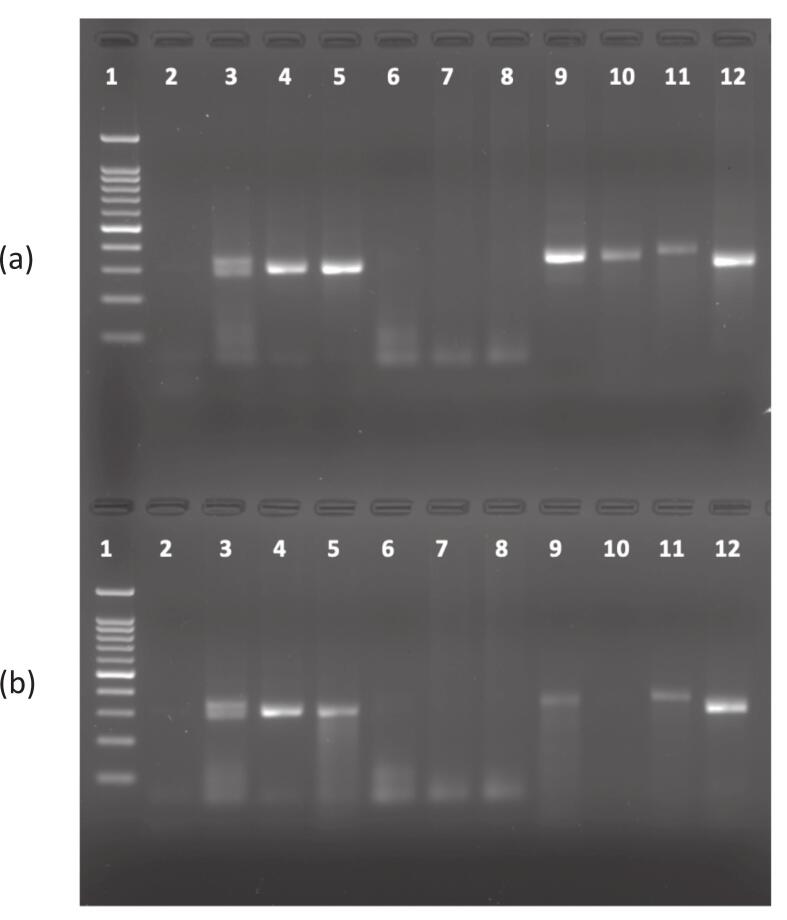
(a) Analysis Conventional PCR using Whole blood (WB) samples and (b) Analysis Conventional PCR using Buccal Swab (BS) samples, shows the PCR amplification results on agarose gel electrophoresis. Lane 1 is a 100 bp marker, Lane 2 is NTC (negative control), and Lanes 3 and 4 are normal female and male controls, respectively. Lane 5 is a positive control for FXS in a female, while Lane 6 is a positive control for FXS in a male. Lanes 7 to 12 contain samples from individuals with Intellectual Disability (ID). The absence of bands in some samples (Lane 7 and 8) suggests a full mutation (CGG repeats > 200), as Conventional PCR cannot amplify large repeat expansions. In panel (b), Lane 10 represents a BS sample that underwent PCR reprocessing. A visible band was initially observed during the first run, but it was no longer clearly visible in the repeated PCR conducted several months after sampling, likely due to the degradation or poor quality of the DNA.

Conventional PCR was performed on all 164 paired samples (whole blood and buccal swab). Two paired samples (ID-008 and ID-159) failed to amplify (no PCR product), indicating presence of full mutation alleles (Fig. 1). Additionally, 3 paired samples (ID-004, ID-005, and ID-007) showed bands higher than the normal control (approximately 350 bp), suggesting presence of grey zone alleles. The remaining 159 paired samples showed bands that aligned with the control band (300 bp), indicating normal alleles.

**Fig. 2:**
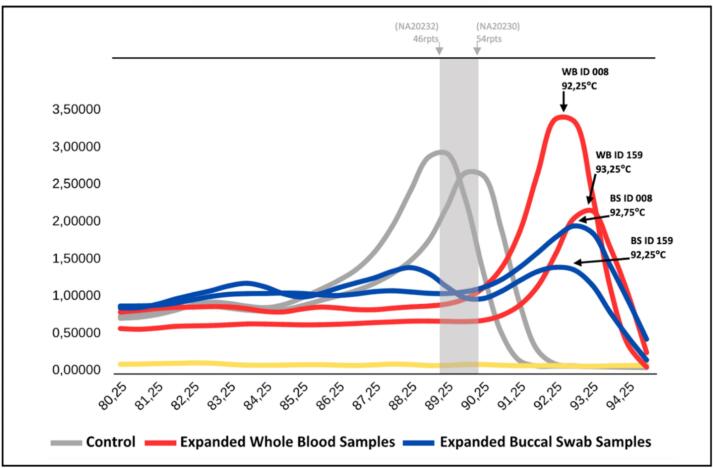
The analysis of the melting curve of TP-PCR MCA showed distinct peaks for WB (whole blood) and BS (buccal swab) samples of ID 008 (92.25 °C and 92.75 °C, respectively) and ID 159 (93.25 °C and 92.25 °C, respectively), indicating expanded CGG repeats, while the normal allele at the cutoff melting temperature (grey).).

**Fig. 3:**
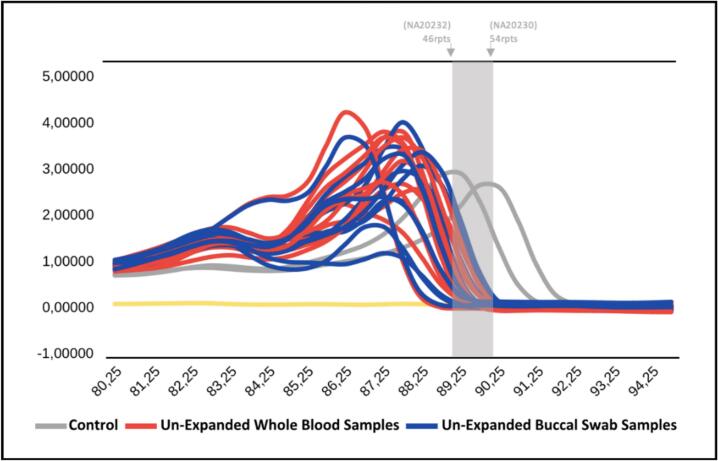
The melting curve analysis of TP-PCR MCA showing unexpanded whole blood (red) and buccal swab samples (blue) for CGG repeats of *FMR1*. Controls are grey, with the baseline for negative control (yellow). Both sample types show consistent melting peak, aligning with reference alleles (46 and 54 repeats), supporting buccal swabs as a reliable noninvasive alternative. (For interpretation of the references to colour in this figure legend, the reader is referred to the web version of this article.)

**Fig. 4:**
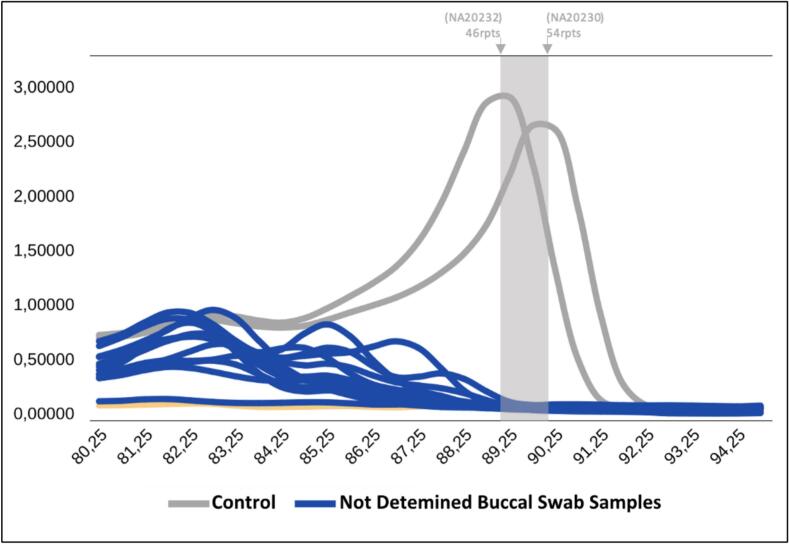
The melting curve analysis of TP-PCR MCA showing buccal swab samples not determined (blue). Distribution curve of the PCR peak from buccal swab samples that were not clearly identified in the analysis. (For interpretation of the references to colour in this figure legend, the reader is referred to the web version of this article.)

A total of 80 samples were selected for TP-PCR MCA form the available 164 paired specimens. Due to cost-effectiveness considerations in our resource-limited setting, and since the main purpose of this study was to determine the prevalence of FXS in Jakarta as well as to compare buccal swab and blood samples (Table 1), the subset was chosen to remain representative of the study samples. Expanded samples are shown in Fig. 2, while unexpanded samples are presented in Fig. 3. The results indicated variations in classification between the two types of samples. For whole blood samples, 78 were negative, 2 were positive, and no intermediate or non-determined results were observed. Meanwhile, for buccal swab samples, 64 were tested negative, 2 were tested positive, 2 were classified as intermediate, and 12 were classified as indeterminate (Fig. 4).

**Table 1:** TP-PCR MCA results comparing whole blood and buccal swab samples for fragile X syndrome detection.

Sample	Negative	Positive	Intermediate	Not determined
Whole Blood	78	2	−	−
Buccal Swab	64	2	2	12

^a^Negative refers to the absence of repeat expansion of CGG, Positive indicates the presence of expansion, Intermediate suggests uncertain classification, and Not Determined represents inconclusive results due to technical limitations.

**Fig. 5:**
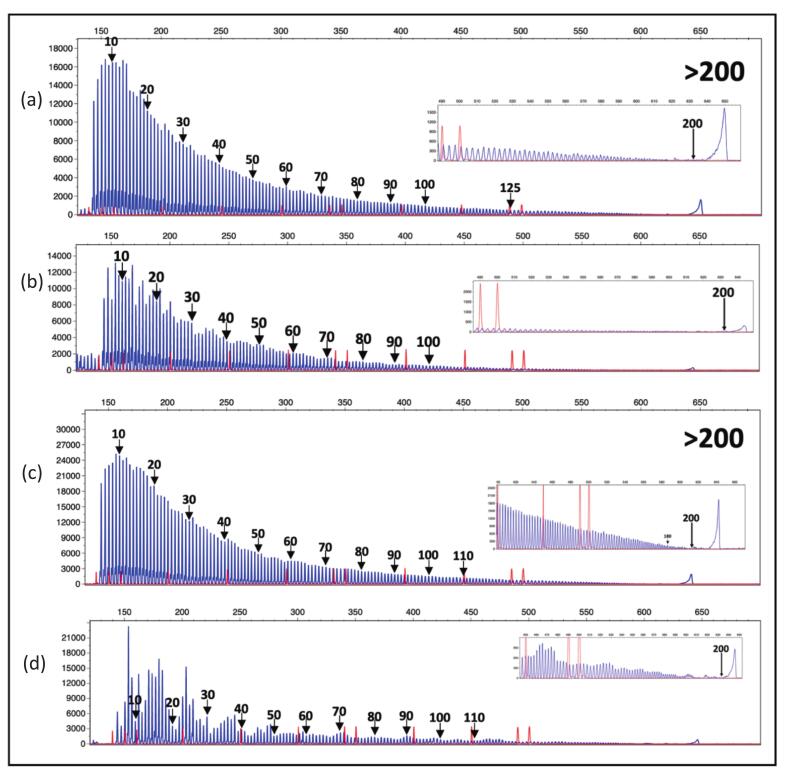
Fluorescent TP-PCR and CE showing both whole blood and buccal swab samples (a) Whole blood of sample ID-008, (b) Buccal swab sample ID-008, (c) Whole blood of sample ID-159 and (d) Buccal swab sample consistently show CGG repeat expansions beyond 200, confirming the presence of a complete mutation.

Sizing confirmation of all expanded samples and selected non-expanded samples was performed using TP-PCR CE. The two paired samples with positive results and three paired samples with negative results from TP-PCR MCA were subjected to size confirmation. Fluorescent TP-PCR CE analysis confirmed CGG repeat expansions > 200 in both expanded paired samples (Fig. 5). Furthermore, the CGG repeat size of the three paired samples with normal allele were confirmed to have repeats < 45, consistent with normal alleles.

**Fig. 6:**
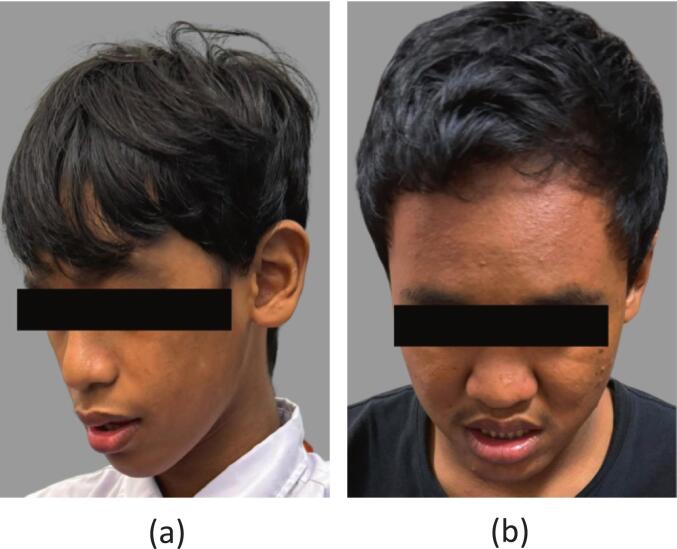
The facial features of full mutation ID 008 (a) and ID 159 (b). FXS clinical stigmata found on both patient such as long face and prominent ears.

The dysmorphological features observed in the two samples with positive results exhibit characteristics commonly associated with FXS. Sample ID 008 (Fig. 6a) presents distinct dysmorphic features, including a prominent forehead, an elongated face, prominent ears, flat feet, and macroorchidism, which is a hallmark physical trait in males with this syndrome. Additionally, this individual demonstrates social interaction deficits, characterized by poor eye contact and a strong tendency to avoid gaze. Meanwhile, sample ID 159 (Fig. 6b) shares several similar features, such as a prominent forehead, an elongated face, prominent ears, flat feet, and poor eye contact. These differences in clinical manifestation may reflect variations in phenotypic expression influenced by genetic and environmental factors.

**The Use of TP-PCR MCA using Two Difference sampling methods**

TP-PCR MCA was performed on 80 whole blood samples to detect CGG repeat expansion in the *FMR1* gene. The results showed that 2 samples (2.5 %) tested positive, while the remaining 78 samples (97.5 %) were negative. These findings indicate that the TP-PCR MCA method can detect CGG repeat expansion with high sensitivity, consistent with previous research by Sihombing et al. (2021), which evaluated the effectiveness of TP-PCR MCA in screening the *FMR1* gene in individuals with intellectual disabilities. The study highlighted that this technique, especially when combined with methylation analysis, can improve accuracy in detecting premutations and full mutations.^16^ Therefore, our findings further support the evidence that TP-PCR MCA is a reliable method for detecting CGG expansions in the context of Fragile X Syndrome (FXS) screening.

**The Use of conventional PCR using Two Difference sampling methods**

Conventional PCR was performed on 164 whole blood samples, yielding 2 full mutation cases, 3 grey zone cases, and 159 normal alleles. The analysis was subsequently conducted on buccal swab samples from the same individuals, producing identical results to those obtained from whole blood samples. Further analysis using Cohen's Kappa coefficient revealed perfect agreement (Kappa = 1.000, 95 % CI: 1.000 to 1.000), indicating a high level of concordance between the two methods. These findings suggest that conventional PCR provides highly reliable results across both whole blood and buccal swab samples, supporting the use of buccal swabs as a viable alternative for genetic screening in FXS detection.

To evaluate the sensitivity and specificity, we ran PCR conventional using 2 positive samples and 78 negative samples which were tested previously using TP-PCR MCA. Conventional PCR results showed a sensitivity of 100.00 % (95 % CI: 15.81 % to 100.00 %) and a specificity of 100.00 % (95 % CI: 95.38 % to 100.00 %). These results highlight the high diagnostic accuracy of conventional PCR in detecting CGG repeat expansions in the *FMR1* gene. However, despite its reliability, conventional PCR may have limitations in detecting mosaicism or accurately sizing large repeat expansions. TP-PCR MCA, on the other hand, offers enhanced resolution and may be more suitable for detecting complex repeat expansion patterns, particularly in cases where mosaicism is suspected.

## 4 Discussion

Conventional PCR cannot detect CGG repeat expansions exceeding 120 repeats, thus missing large premutation and full mutation alleles. To improve accuracy, further analysis was conducted using TP-PCR, which uses three primers to amplify larger expansions. The resulting TP-PCR products were analyzed using MCA to assess melting profiles and detect CGG repeat expansions.

Our study confirmed that DNA extracted from buccal swabs had lower concentrations than blood-derived DNA. However, Woo et al. (2007) showed that proper collection and storage can yield DNA from buccal swabs with 98.8 % concordance to blood, suggesting that technical factors likely influence variability more than sample type.^17^ Despite the lower yield, conventional PCR in our study still successfully amplified buccal DNA and detected CGG expansions. This supports buccal swabs as a viable alternative for genetic testing. This study emphasizes the diagnostic performance of buccal versus blood-derived DNA for *FMR1* analysis using PCR-based methods.

Our findings show that conventional PCR maintained high sensitivity and specificity for detecting CGG expansions up to 120 repeats in both blood and buccal swab samples. In contrast, TP-PCR MCA produced more indeterminate results in buccal swabs, suggesting the need for refinement when using buccal-derived DNA. These findings highlighted notable differences in detection outcomes between whole blood and buccal swab samples, when using TP-PCR MCA. This may reflect limitations in the quality and/or quantity of DNA extracted from buccal swabs, that potentially affecting assay performance. Despite this, Zhao et al. (2017) confirmed TP-PCR MCA’s high sensitivity across various DNA sources, including buccal swabs.^18^ Rajan-Babu et al. (2022) similarly demonstrated its reliability in detecting low-level mosaicism across a broad range of DNA concentrations. ^15^ These discrepancies likely stem from DNA quality or contaminants, not the method itself. Incorporating internal size controls, as recommended by Rajan-Babu et al., could enhance result interpretation and reduce indeterminate calls in buccal swab analysis.

Our study identified three samples with bands above the normal control, interpreted as grey zone alleles, suggesting that conventional PCR can detect repeat expansions but has limitations in classifying specific allele types. A major challenge is detecting mosaicism, especially in individuals with both premutation and full mutation alleles. Mosaicism has been reported in approximately 12–41 % of individuals with full mutations, and conventional PCR often fails to identify these cases.^18^ The prevalence of FXS in the Indonesian ID population has been reported at 0.9 %–1.9 %, and 6.15 % among children with ASD.^7^ In our study, two out of 164 children with ID were diagnosed with FXS (1.22 %), consistent with previous findings and supporting the need for routine screening and cascade testing, especially in cases of unexplained ID.

Our findings suggest that conventional PCR detection is robust and reproducible, but TP-PCR MCA requires optimization for buccal swabs, potentially through the enhancement of DNA extraction protocols to improve yield and purity, increasing DNA input concentration in MCA reactions, and exploring alternative analytical methods such as digital PCR or next-generation sequencing (NGS) to validate MCA results. Rajan-Babu et al. (2022) demonstrated that an improved msTP-PCR MCA assay enabled the selective and reliable identification of actionable *FMR1* genotypes, detecting CGG repeat expansions with high sensitivity and specificity in peripheral blood-derived DNA. Their study also highlighted that methylation-specific TP-PCR MCA can identify premutation/full mutation mosaicism, which is relevant to refine diagnostic strategies for FXS and related conditions.^15^, ^19^ However, further studies are needed to determine whether this method can be reliably adapted for buccal swab-derived DNA.

Buccal swabs have become a promising alternative to blood samples for genetic testing due to their noninvasive, simple, and painless collection, making them especially suitable for pediatric patients, individuals with intellectual disabilities, and those with phlebotomy-related anxieties.^20^ Unlike venous blood collection, buccal swabs require minimal infrastructure and trained personnel, which is particularly advantageous in low- to middle-income countries. Our study supports these benefits, showing that DNA from buccal swabs despite lower concentrations was sufficient for conventional PCR amplification. However, for more complex assays such as TP-PCR MCA, optimization of DNA extraction and sample collection techniques remains necessary. This aligns with previous studies recommending refinement of buccal DNA processing to enhance diagnostic reliability. Nonetheless, we encountered challenges during sampling in children with disabilities, including resistance and variable cooperation, which affected DNA yield and quality. These findings highlight the importance of developing adaptive, child-friendly collection methods to ensure consistent and high-quality DNA for genetic testing in special needs populations.

In addition to being noninvasive, buccal swab collection is quick and straightforward, requiring no specialized skills or equipment. It can be administered independently or performed by nonmedical personnel, making it an excellent choice for field studies, remote tests, and community genetic screening programmes.^21^ The cost-effectiveness of buccal swabs when used with conventional PCR methods is a significant advantage, as they eliminate the need for phlebotomy, syringes, and specialized transport conditions such as cold chain logistics. This reduction in infrastructure and labour costs makes buccal swabs a practical and economical alternative, particularly in resource-limited settings and areas with limited access to laboratory infrastructure.^20^, ^22^ Our study supports these advantages, as buccal swabs provided an accessible and feasible method for collecting DNA samples from children with intellectual disabilities.

Buccal swabs are highly suitable for large-scale genetic screening programs, as they facilitate rapid and high-throughput sample collection. Their ease of transport and storage makes them also ideal for epidemiological studies and population-based genetic research.^21^ DNA extracted from buccal swabs is stable at room temperature for extended periods, reducing the need for cold storage during transport. This is particularly beneficial for studies conducted in remote areas or low-resource settings where maintaining a cold chain is challenging.

This was the first screening conducted in Jakarta, the capital city of Indonesia, which has a population of approximately 11 million, for this purpose. This finding suggests the presence of FXS in these individuals, reinforcing the importance of genetic screening in early diagnosis and intervention planning for the next generation in the family. The identification of these cases underscores the necessity of implementing broader screening programs, particularly in populations with intellectual disabilities, to facilitate timely medical and educational support.

## 5 Conclusion

Buccal swabs provide a feasible and advantageous alternative to blood samples for genetic testing, particularly in noninvasive, cost-effective, and scalable settings. This study marks the first implementation of FXS screening in Jakarta using conventional PCR followed by TP-PCR MCA, a strategy that balances affordability and diagnostic accuracy in a country with limited access to advanced molecular facilities. We propose a two-step molecular approach where conventional PCR serves as an initial screening method due to its simplicity and low cost. Samples with CGG repeat expansions or inconclusive results can then undergo TP-PCR MCA for clarification. In cases where mosaicism or full expansions are suspected, Southern blot or methylation-specific PCR may be required for confirmation. This model could be applied not only to FXS but also adapted for other trinucleotide repeat disorders, especially in resource-limited environments. Integrating such a protocol into clinical practice may enhance early diagnosis, inform reproductive decisions, and support genetic counseling and cascade testing for affected families.

## CRediT authorship contribution statement

**Siti F. Aulia:** Writing – review & editing, Writing – original draft, Visualization, Software, Resources, Project administration, Methodology, Formal analysis, Data curation, Conceptualization. **Mentari Amir:** Writing – review & editing, Writing – original draft, Visualization, Software, Resources, Project administration, Methodology, Formal analysis, Data curation, Conceptualization. **Intan Razari:** Visualization, Software, Project administration, Methodology, Conceptualization. **Kinasih Prayuni:** Writing – review & editing, Visualization, Software, Project administration, Methodology. **Wan Nedra:** Writing – review & editing, Conceptualization. **Ndaru A. Damayanti:** Writing – review & editing, Conceptualization. **Nurmayani Irwandi:** Resources, Formal analysis. **Ahmad Utomo:** Writing – review & editing, Validation, Methodology, Funding acquisition, Formal analysis, Conceptualization. **Vivienne J. Tan:** Writing – review & editing, Validation. **Samuel S. Chong:** Writing – review & editing, Validation. **Sultana M.H. Faradz:** Writing – review & editing, Writing – original draft, Validation, Supervision, Methodology, Investigation, Funding acquisition, Formal analysis, Data curation, Conceptualization.

## Declaration of competing interest

The authors declare the following financial interests/personal relationships which may be considered as potential competing interests: Sultana M.H. Faradz reports financial support was provided by Ministry of Research and Technology National Research and Innovation Agency. If there are other authors, they declare that they have no known competing financial interests or personal relationships that could have appeared to influence the work reported in this paper.

## References

[1] Acero-Garcés D.O., Saldarriaga W., Cabal-Herrera A.M., Rojas C.A., Hagerman R.J. (2023). Fragile X Syndrome in children. Colomb Med..

[2] Cartwright L., Scerif G., Oliver C. (2025). Genetic determinants of longitudinal behavioural trajectories in rare conditions: the case of fragile X syndrome. Behav Brain Res..

[3] CDC: Data and Statistics on Fragile X Syndrome. U.S Center For Disease Control and Prevention. 2024. Accessed November 28, 2024. https://www.cdc.gov/fragile-x-syndrome/data/index.html.

[4] Spector E., Behlmann A., Kronquist K., Rose N.C., Lyon E., Reddi H.V. (2021). Laboratory testing for fragile X, 2021 revision: a technical standard of the American College of Medical Genetics and Genomics (ACMG). Genet Med..

[5] Reiss S., Zalles L., Gbekie C., Lozano R. (2021). Identity and reproductive aspects in females with fragile X syndrome. Women’s Health Reports..

[6] Ma Y., Wei X., Pan H. (2019). The prevalence of CGG repeat expansion mutation in FMR1 gene in the northern Chinese women of reproductive age. BMC Med Genet..

[7] Sihombing N.R.B., Winarni T.I., Utari A., Van B.H., Hagerman R.J., Faradz S.M.H. (2021). Surveillance and prevalence of fragile X syndrome in Indonesia. Intractable Rare Dis Res..

[8] Protic D.D., Aishworiya R., Salcedo-Arellano M.J. (2022). Fragile X syndrome: from molecular aspect to clinical treatment. Int J Mol Sci..

[9] Ciobanu C.G., Nucă I., Popescu R. (2023). Narrative review: update on the molecular diagnosis of fragile X syndrome. Int J Mol Sci..

[10] Lyons J.I., Kerr G.R., Mueller P.W. (2015). Fragile X syndrome scientific background and screening technologies. J Mol Diagnost..

[11] Tan V.J., Lian M., Faradz S.M.H., Winarni T.I., Chong S.S. (2018). A single common assay for robust and rapid fragile X mental retardation syndrome screening from dried blood spots. Front Genet..

[12] Fu Y.H., Kuhl D.P.A., Pizzuti A. (1991). Variation of the CGG repeat at the fragile X site results in genetic instability: resolution of the Sherman paradox. Cell..

[13] Chen Q., Wang Q.Q., Cai B.Z., Ren X.J., Zhang F., Zhang X.J. (2017). Analysis of the fragile X mental retardation 1 Premutation in Han Chinese women presenting with primary ovarian insufficiency. Reproduct Dev Med..

[14] Rajan-Babu I.S., Law H.Y., Yoon C.S., Lee C.G., Chong S.S. (2015). Simplified strategy for rapid first-line screening of fragile X syndrome: closed-tube triplet-primed PCR and amplicon melt peak analysis. Expert Rev Mol Med..

[15] Rajan-Babu I.S., Phang G.P., Law H.Y., Lee C.G., Chong S.S. (2022). High-throughput methylation-specific triplet-primed PCR and melting curve analysis for selective and reliable identification of actionable FMR1 genotypes. J Mol Diagnost..

[16] Sihombing N.R.B., Cai S., Wong D.P.W. (2021). Repeat expansion and methylation-sensitive triplet-primed polymerase chain reaction for fragile X mental retardation 1 gene screening in institutionalised intellectually disabled individuals. Singapore Med J..

[17] Woo J.G., Sun G., Haverbusch M. (2007). Quality assessment of buccal versus blood genomic DNA using the Affymetrix 500 K GeneChip. BMC Genet..

[18] Zhao M., Cheah F.S.H., Chen M., Lee C.G., Law H.Y., Chong S.S. (2017). Improved high sensitivity screen for Huntington disease using a one-step triplet-primed PCR and melting curve assay. PLoS One..

[19] Liang Q., Liu Y., Liu Y. (2022). Comprehensive analysis of fragile X syndrome: Full characterization of the FMR1 locus by long-read sequencing. Clin Chem..

[20] Sy A., McCabe L., Hudson E. (2023). Utility of a buccal swab point-of-care test for the IFNL4 genotype in the era of direct acting antivirals for hepatitis C virus. PLoS One..

[21] Duffy D., Mottez E., Ainsworth S. (2017). An in vitro diagnostic certified point of care single nucleotide test for IL28B polymorphisms. PLoS One..

[22] Kamga K.K., Nguefack S., Minka K. (2020). Cascade testing for Fragile X syndrome in a rural setting in cameroon (Sub-Saharan Africa). Genes (basel)..

